# The Impact of Active Augmented Reality Games on Physical Activity and Cognition Among Older Adults: Feasibility Study

**DOI:** 10.2196/73221

**Published:** 2025-10-03

**Authors:** Amy Shirong Lu, Bhagyashree Parkar, Shiveshchandra Gupta, Sierra Hall, Dominika M Pindus, Arthur F Kramer

**Affiliations:** 1 Health Technology Lab, Department of Communication Studies, College of Arts, Media, and Design, Department of Public Health Science and Health Sciences, Bouvé College of Health Sciences, Northeastern University, 360 Huntington Ave 212A Lake Hall, Boston, MA, 02115, United States, 1 6173737839; 2 Department of Kinesiology and Community Health, Institute of Applied Life Sciences, University of Massachusetts Amherst, Amherst, MA, United States; 3 Beckman Institute for Advanced Science and Technology, University of Illinois, Urbana, IL, United States

**Keywords:** active augmented reality, physical activity, active video games, cognitive function, older adults, feasibility study, health technology, AAR, PA, AVG

## Abstract

**Background:**

Physical activity (PA) enhances physical health as well as cognitive and brain health, yet motivating older adults to initiate and sustain PA remains challenging, a difficulty exacerbated by the COVID-19 pandemic. Active augmented reality (AAR) games, integrating digital gameplay with real-world physical movement, provide an enjoyable and accessible means for PA promotion among older adults in independent living environments, mitigating barriers such as poor weather and unfavorable neighborhood environments. However, limited research has explored the feasibility and impact of AAR interventions in this population.

**Objective:**

This feasibility study examines the acceptability, safety, and preliminary effects of AAR games to enhance PA levels and cognitive functions among older adults, as well as their user experiences. We also examined the practicality of home-based AAR gameplay using minimal equipment and constrained physical space.

**Methods:**

Sixteen independent-living older adults aged 65‐85 years (mean 74.6, SD 3.73) participated in a single-session AAR intervention using the *Active Arcade* game set by playing four 10-minute AAR games. PA levels were assessed using ActiGraph wGT3x-bt accelerometers and Polar H10 heart rate monitors. Cognitive function was evaluated pre- and post-gameplay using NIH Toolbox’s visual reasoning test and Flanker inhibitory control and attention tests. Surveys of PA intention and motivation as well as the gaming experience questionnaire, along with semistructured interviews, were conducted afterwards, providing both quantitative and qualitative insights into the feasibility and appeal of AAR gameplay from the target population.

**Results:**

All participants completed the study protocol without adverse events, demonstrating high feasibility and acceptability. Participants engaged in moderate-to-vigorous PA during 20%‐30% of the gameplay, as measured by accelerometers and heart rate monitors. Of the 16 participants, 7 were taking beta blockers. The mean values of average %HRMax suggest that those not on beta blockers generally met the moderate-intensity threshold, whereas those on beta blockers tended to fall slightly below it. Cognitive assessments revealed significant improvements in visual reasoning postintervention, with the effect sustained after adjustment for age and education (*P*=.03), suggesting potential cognitive benefits from a single bout of AAR gameplay. Survey responses indicated high levels of PA intention (mean 4.15/5, SD 0.59), motivation (mean 5.67/7, SD 1.24), high positive affect (mean 4.35/5, SD 0.80), and low negative affect (mean 1.30/5, SD 0.46) associated with AAR gameplay. Around 75% of gameplay occurred within a 4×4 ft area (mean 29.77/40 min, SD 2.46), indicating suitability for home environments. Thematically analyzed interview feedback emphasized participants’ enjoyment, ease of use, desire for progressive difficulty, and the need to cater to diverse physical abilities and individual preferences.

**Conclusions:**

AAR games are a feasible, accessible, and enjoyable alternative for PA and cognitive engagement among older adults. Future research should investigate the long-term effects, sustainability, and broader applicability of AAR interventions to fully realize their potential in aging populations.

## Introduction

More than one-fifth of the US residents will be over the age of 65 years by 2030 [1]. This significant demographic shift will increase both the relative and absolute number of older adults, presenting challenges across various aspects of health [2]. Physical activity (PA) levels decrease significantly with age [3,4], and low PA is associated with numerous adverse outcomes such as high blood pressure, frailty, increased fall risk, depression, and cognitive decline [5-7]. Maintaining PA levels and cognitive function is pivotal for functional independence, effective communication, and overall quality of life [8-10]. There is a close connection between the cognitive well-being and older adults’ ability to manage daily tasks, with cognitive decline significantly impairing their ability for independent living [11-13].

According to the United Nations, one of the sustainable development goals is to ensure healthy lives and promote well-being for all ages [14]. Moderate-to-vigorous PA (MVPA) is one of the most effective strategies to achieving these goals. It has been shown to decrease morbidity, prevent cognitive decline, and reduce the risk of mild cognitive impairment and Alzheimer disease and related dementias [15-18]. In fact, many forms of PA enhance physical, cognitive, and brain health and serve as a protective factor against future cognitive decline [19,20]. PA also improves cardiorespiratory fitness, reduces frailty and fall risk, and increases quality of life [21-23]. Additionally, PA has demonstrated an antidepressant effect in older adults, further contributing to their emotional and mental well-being [24].

The World Health Organization recommends that adults, including older adults, engage in at least 150 minutes of moderate-intensity or 75 minutes of vigorous-intensity PA per week [25]. However, motivating older adults to initiate and sustain PA remains challenging, exacerbated by the COVID-19 pandemic, which has created additional barriers to PA [26]. Even after the pandemic has passed, its negative impact on older adults persists, contributing to declines in PA levels, mobility, and overall well-being [27]. Common obstacles include low motivation, limited access to facilities, fear of injury or falls, and a lack of knowledge about proper exercise techniques [28]. Addressing these challenges is critical to providing older adults with opportunities to increase PA levels, promoting their physical and cognitive health while fostering overall well-being.

The American College of Sports Medicine has identified the integration of technology to achieve PA goals as “the future of fitness,” with exercise programs for older adults ranking third on their list of trends for 2025 [29]. Active video games (AVG), or any video game that requires PA as part of gameplay, have the potential to overcome traditional barriers to PA for older adults. Extended reality technologies, including virtual reality (VR) and augmented reality (AR), have shown promise in promoting PA and enhancing cognitive functioning in older adults [30]. Active augmented reality (AAR) games, a subset of AVG, leverage AR technologies to incorporate active gameplay. Unlike VR, AR integrates digital graphics and information into the user’s physical environment, creating an immersive and interactive experience that blends the real and virtual worlds [31].

AR technology has experienced rapid growth, with a projected market value exceeding US $635 billion by 2033 [32,33]. In 2022, over 70% of Americans believed that AR would become an integral part of everyday life, with 89% viewing it as beneficial [34]. Notably, the baby boomer generation has shown a surprising openness to exploring extended reality technologies. A 2023 survey revealed that 70% of baby boomers expressed interest in trying AR/VR technology, suggesting its potential for adoption among older adults [35]. Indeed, older adults have demonstrated positive attitudes and high interest in AR [36-39]. Active AR, or AAR interventions, have been successfully implemented in older adults for various health-related purposes, including improving balance [40] and reducing muscle loss [41]. AAR has also been well received by those with Alzheimer disease and related dementias [42] and Parkinson disease [43], indicating its adaptability across different aging-related conditions.

AAR games integrate real-world movements to enhance physical coordination while keeping users engaged through interactive, immersive experiences. By combining physical exercise with cognitive challenges, AR-based game interventions provide a dual benefit, promoting both physical and cognitive health [44]. While these initial findings are promising, there remains a significant gap in research on AAR’s role in promoting PA and preventing cognitive decline. Understanding older adult usage of AAR is crucial for maximizing its potential as a health-promoting intervention.

AAR could serve as an effective, enjoyable, and accessible method for promoting PA motivation and adherence among older adults, mitigating traditional PA barriers such as poor weather and unsafe neighborhood environments. While older adults have historically been underrepresented in digital design, which partly contributes to their lower utilization of AR devices [45], they have shown a growing enthusiasm for AR technologies [33]. AAR games are increasingly gaining popularity as a means of engaging older adults in PA [46,47]. Commercially available platforms, such as Nintendo Wii and Microsoft Kinect, have introduced AAR-based games that enable users to engage in interactive exercise experiments from the comfort of their homes. These systems offer games designed to accommodate varying levels of difficulty and skill, making them a versatile option for older adults seeking to integrate PA into their daily routines. Moreover, AR technology extends beyond physical exercise, contributing to the psychological and social well-being of older adults. Research indicates that AR applications create enjoyable and engaging experiences, which can enhance motivation and promote adherence to exercise routines [30]. Additionally, the immersive nature of AR fosters mental engagement, helping older adults combat loneliness and social isolation [30].

As AR technology continues to evolve, further research is needed to address existing challenges and investigate the long-term benefits of AR interventions on cognitive health and physical abilities in older adults. This feasibility study seeks to bridge this research gap by examining the positive effects of AR games on PA and cognition in older adults. The study employs a mixed-method design with pre- and postintervention assessments, integrating both quantitative and qualitative research methods to evaluate the impact of AAR games on PA levels and cognitive functioning in older adults. The following research questions (RQs) guide this investigation:

RQ1: Can AAR induce MVPA among older adults?

RQ2: Will AAR improve older adults’ PA intention?

RQ3: Can AAR improve cognitive outcomes among older adults?

RQ4: What are older adults’ cognitive and affective responses to the AAR gaming experience?

## Methods

### Ethical Considerations

The Northeastern University Institutional Review Board approved the study (#23-09-28). All participants signed written informed consent. Each participant completed the protocol behind a closed door, and the study data were deidentified before being analyzed. Each participant received a US $100 gift card at the end of the study session.

### Sample

Participants were recruited through web advertisements and on-campus posters at the first author’s institution. Additionally, potential participants were identified from the IGNITE (Investigating Gains in Neurocognition in an Intervention Trial of Exercise) [48] trial database and contacted by phone. The inclusion criteria targeted adults aged 65‐85 years, ensuring an equal gender distribution, English language proficiency, absence of cognitive impairments, and sufficient physical ability to complete the study protocol (ie, without significant balance or mobility issues). More specifically, all participants completed a comprehensive neuropsychological evaluation, including the Montreal Cognitive Assessment, and all participants passed without being classified as cognitively impaired [49]. Exclusion criteria included any history of acute or chronic physical, cognitive, or mental health conditions that could interfere with participation in exercise. Approval from participants’ primary care providers was also required. To control for potential bias, only individuals who had never played the AAR games selected for the study were invited to participate.

Approximately 160 individuals were contacted by phone, with 65 expressing interest in the study. Of these, 39 older adults underwent screening using the Physical Activity Readiness Questionnaire [50] and the Late Life Function and Disability Screening Function and Disability Component [51]. A total of 17 respondents passed the screening, and 16 participants were included in the study.

### AAR Game

For this project, we selected *Active Arcade* (Nex, San Jose, CA) as the AAR game set. As one of the newer AR-based AVGs, *Active Arcade* is powered by proprietary computer vision technology used in pro sports and is freely available on iOS [52] and Android [53] stores. *Active Arcade* tracks body movements via a smartphone or tablet camera, requiring no wearables. It offers full-body games playable solo or with others, making it accessible across age and fitness levels. Its simple setup suits home-based use. A 2022 Apple Design Award finalist, the app has logged over 100 million play sessions since its 2021 launch [54,55]. It is compatible with smartphones and tablets on both major operating systems and can be displayed on any TV using an HDMI adapter [55].

### General Procedures

Upon arrival, participants were provided with written informed consent before completing an online questionnaire (see Multimedia Appendix 1) to record demographic information. Research assistants (RAs) measured each participant’s height using a stadiometer (ShorrBoard, Weight and Measure, LLC, Olney, MD) and weight with a calibrated scale (SECA 813, SECA Inc., Chino, CA). To ensure focus during the session, the RAs instructed participants to set their personal mobile devices to “Do Not Disturb” mode.

An accelerometer (wGT3x-bt; ActiGraph, Pensacola, FL) was attached to each participant’s nondominant hip, and a heart rate (HR) band (Polar H10; Polar, Kempele, Finland) was secured to their chest. Baseline resting HR and rate of perceived exertion (RPE) were recorded using the Borg Category-Ratio (CR-10) [56]. Participants then completed two cognitive tests from the NIH Toolbox, the visual reasoning test, and the Flanker inhibitory control and attention test, before receiving gameplay instructions from the RAs. The NIH Toolbox is a validated, standardized battery developed by the National Institutes of Health to measure neurological and behavioral function across the lifespan [57]. For this study, we selected two specific tests because they target cognitive domains (fluid reasoning and executive function) that have shown associations with PA in older adults [58,59]. Visual reasoning assesses problem-solving and visuospatial skills, while the Flanker task measures attention and inhibitory control. Together, they offer a concise but meaningful assessment of the cognitive processes most likely to be influenced by interactive, movement-based AAR gameplay.

**Figure 1.**
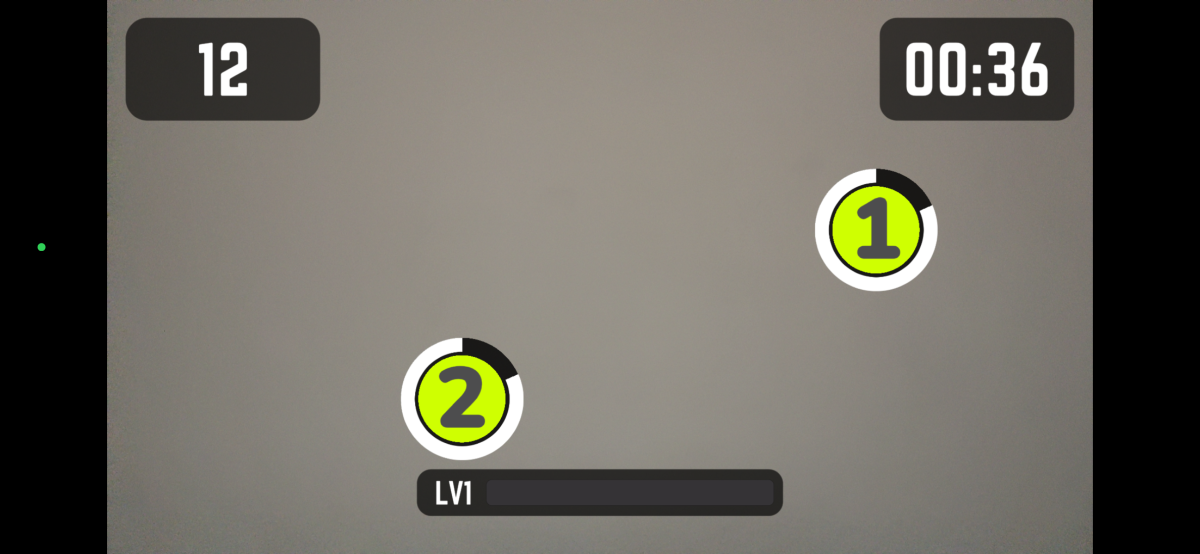
Reaction Flow.

**Figure 2.**
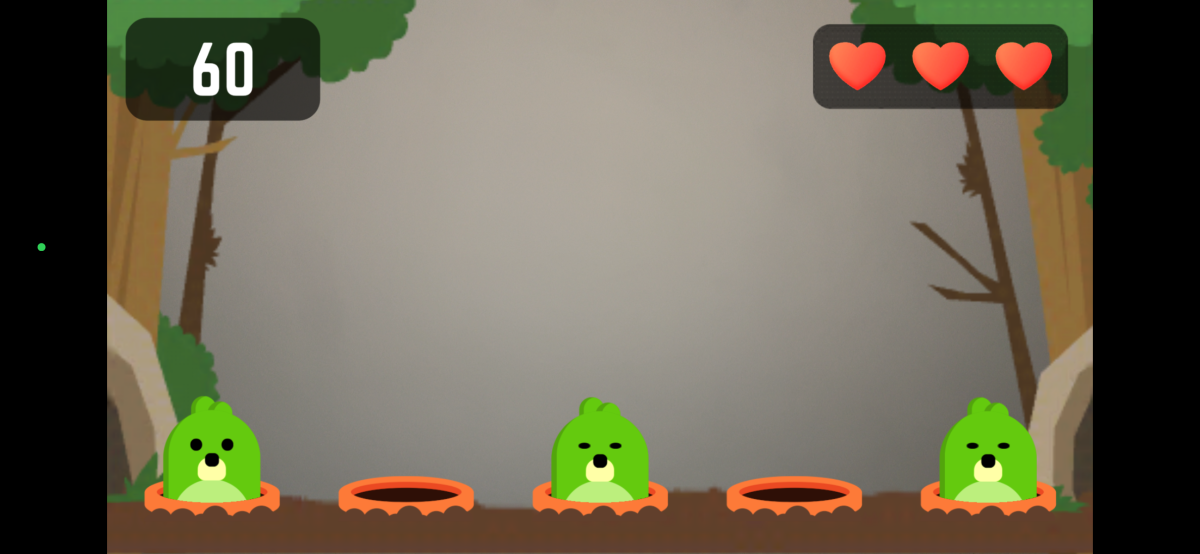
Whack a Mole.

**Figure 3.**
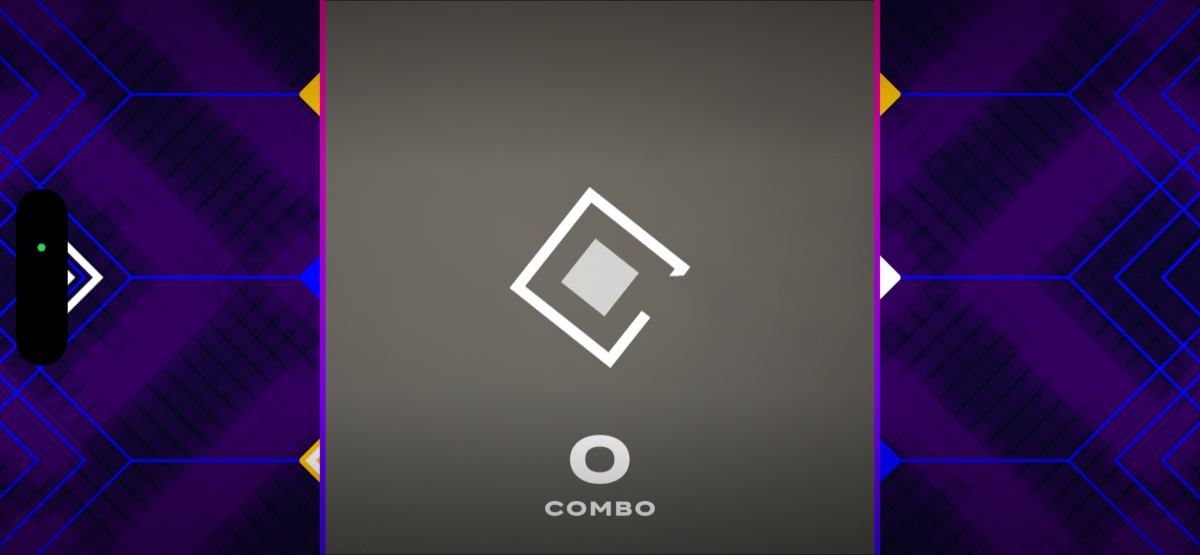
Super Hits—Catch.

**Figure 4.**
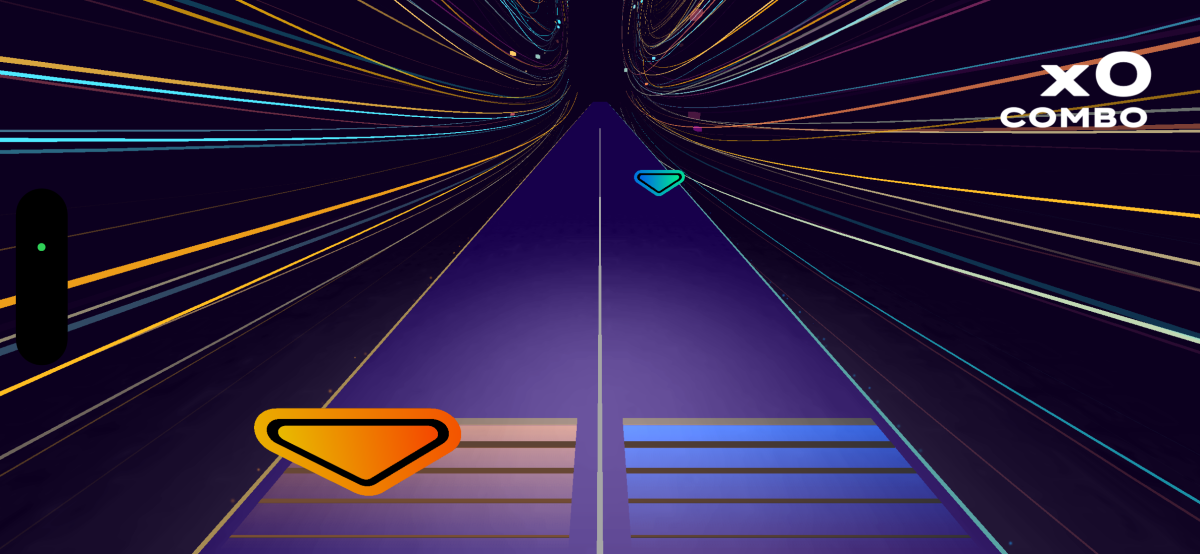
Super Hits—Slash.

Participants engaged in four 10-minute *Active Arcade* AR mini-games, interspersed with rest intervals, while HR was continuously monitored. The games were randomly assigned in blocks with progressively increasing intensities. The *Active Arcade* game includes a variety of mini games; for this study, participants played *Reaction Flow* (Figure 1), *Whack a Mole* (Figure 2), and two modes of *Super Hits* [Catch (Figure 3) and Slash (Figure 4)], totaling 40 minutes of gameplay (10 min per game). We did not modify any part of the game, as the *Active Arcade* game set was designed for all ages and ability levels, with games accessible to both children and older adults. These four games, as well as their order of presentation, were pretested and selected by two additional older adults meeting the same inclusion criteria and by lab staff experienced in AVG research, using the same PA measurement protocol, to ensure that the level of difficulty increased progressively. The gameplay took place in a square-shaped space divided into inner (4×4 sq ft), middle (6×6 sq ft), and outer (8×8 sq ft) zones. Games began only when participants’ HR was within their resting HR range. After gameplay, participants repeated the NIH Toolbox cognitive tests to assess post-gameplay cognitive performance.

After gameplay, participants completed surveys assessing intentions, motivations, and gaming experience. The session concluded with audio- and video-recorded interviews exploring participants’ gameplay experience and attitudes toward AR.

### Physical Activity Assessments

To assess PA, an ActiGraph wGT3x-bt accelerometer (ActiGraph, Pensacola, FL) was attached to each participant’s nondominant hip at a 60-Hz sampling rate, recording three-axis movement (anterior-posterior, vertical, and medial-lateral) [60-62]. The RAs documented the start and end times of each 10-minute gameplay period, using these timestamps to identify PA data for each game session. Data were downloaded via ActiLife software (version 6.13.2) and processed in 1-second epochs, a resolution appropriate for capturing short bursts of PA [63]. Activity counts from all three axes and vector magnitude (VM) were extracted, and validated cut-point sets for older adults [64] were applied to classify PA into light (<2690 VM), moderate (≥2,690 to <6,166 VM), and vigorous (≥6166 VM) (VPA) activity levels [65].

A Polar HR monitor (Polar, Kempele, Finland) was used to continuously track PA intensity relative to each participant’s age-predicted maximum HR [66]. PA intensity was calculated using the HR reserve (HRR) formula [HRR=maximum HR (220-age)–resting HR]. Intensity thresholds were defined as light (<40% HRR), moderate (40%‐59% HRR), and vigorous PA (60%‐89% HRR) based on the American College of Sports Medicine guidelines [67]. Individualized cutoff points were applied to determine time spent at each intensity level, with MVPA estimated calculated as the sum of moderate and vigorous PA durations. HR monitors provide a relative estimate of PA intensity while accelerometers will offer an absolute estimation, which is crucial for designing group-based intervention studies due to ease of use in free-living environments [68-71].

### Cognitive Assessments

Cognitive function was assessed using two NIH Toolbox (version 3) [72] tests: the visual reasoning test and the Flanker inhibitory control and attention test. The visual reasoning test is an executive function assessment that evaluates nonverbal and visual reasoning, requiring participants to select the response option that is most like or missing from the target images displayed at the top of the screen. The Flanker inhibitory control and attention test measures inhibitory control and attention, where participants must focus on a particular stimulus while ignoring distracting flanking stimuli. See Multimedia Appendix 2 for screenshots of both tests.

These assessments were administered twice—before and after the gameplay—to evaluate cognitive changes. For post-gameplay assessments, RAs ensured that each participant’s HR returned to within waited for each participant’s HR to drop to within 10 beats per minute (BPM) of their resting HR before initiating the cognitive tests, minimizing potential influences of elevated HR on cognitive performance.

### Questionnaires

Three surveys were used to assess participants’ PA intentions, motivations, and gaming experience: (1) The AAR/PA Intention Scale [73] evaluates attitudes toward PA, perceived behavioral control, and intention to engage in PA. (2) The AAR/PA Motivation is measured by the PA Enjoyment Scale [74]. (3) The Game Experience Questionnaire (GEQ) [75] is a 33-item scale that evaluates gaming experience across multiple dimensions, including competence, immersion, and emotional responses. These validated instruments provide a comprehensive assessment of participants’ engagement with the AAR-based PA and the factors influencing their motivation and experience.

### Interviews

Before leaving the experimental session, participants took part in interviews to share their experiences with AAR games, including their overall impressions and interest in continued gameplay. RAs conducted the interviews using standardized questions that had been previously used in similar studies to ensure consistency and comparability across participants [76,77].

### Data Analyses 

Quantitative data were analyzed using SPSS V.29 (IBM, SPSS Inc., Chicago, IL), with paired-sample *t* tests conducted to assess changes in PA levels and cognitive function. Data are presented as means and standard deviations. All survey questionnaires were scored and analyzed according to their original instructions. The AAR/PA Intention Scale (Cronbach *α*=0.96) [73] and the PA Enjoyment Scale (Cronbach *α*=0.86) [74] were calculated by averaging their respective items. All GEQ-derived variables were scored and analyzed per the developer’s recommendation [75], with subscales excluded from averaging if a Cronbach α was below 0.6 [78]. Qualitative interview data were examined through thematic analysis to identify central themes and patterns in participants’ experiences.

## Results

### Demographics

The average (SD) age of participants was 74.6 (3.73) years. The majority of participants were right-handed (15/16). Racial distribution was equal, with 8 participants identifying as White and 8 as Black; notably, the majority of White participants were male (5 of 8), whereas the majority of Black participants were female (6 of 8). Education levels varied, with the largest proportion holding a Bachelor’s degree (31.25%; Table 1).

**Table 1.** Demographics table, expressed as means and standard deviations.

Characteristic	Overall	Female	Male
Number of participants, n (%)	16	9 (56.25)	7 (43.75)
Age (years), mean (SD)	74.6 (3.73)	74.22 (2.58)	75.14 (5.04)
Height (cm), mean (SD)	168.87 (10.12)	164 (6.5)	175.14 (10.88)
Weight (kg), mean (SD)	84.72 (13.14)	81.40 (12.18)	88.98 (14.02)
Dominant hand, n	Right = 15; Left = 1	Right = 9; Left = 0	Right = 6; Left = 1
Right	15	9	6
Left	1	0	1
Race, n			
White	8	3	5
Black	8	6	2
Education level, n (%)			
High school graduate	3 (18.75)	2	1
Professional degree	3 (18.75)	3	0
Bachelor’s	5 (31.25)	2	3
Master’s	2 (12.5)	2	0
Doctorate	3 (18.75)	0	3

The most prevalent health conditions among participants included high blood pressure and hypertension, with commonly prescribed medications such as amlodipine, losartan, and lisinopril. Statin therapy, including atorvastatin and rosuvastatin, was widely used for cholesterol management. Several participants have histories of significant cardiovascular events, including heart attacks, arrhythmias, and coronary artery stent placements. For instance, one participant experienced a heart attack 6 years ago, while another had stents inserted into the right coronary artery. Other common conditions include osteoarthritis, osteoporosis, and hypothyroidism, along with reports of past cancer diagnoses and respiratory issues. Notably, seven of the 16 participants reported taking beta-blockers, which could lead to underestimation of HR-based PA measurements [79].

### Physical Activities and Zone of Exercise

Table 2 presents the time spent in various PA intensities—light, moderate, and vigorous—as recorded by the ActiGraph and Polar devices across four different games [*Reaction Flow*, *Whack A Mole*, *Super Hits* (*Catch*), and *Super Hits* (*Slash*)]. Although, as expected, vigorous PA levels were relatively low across both devices and all games, each game successfully induced, on average, 20%‐30% MVPA among participants.

**Table 2.** Time spent in minutes in various PA levels using accelerometer and HR monitors, expressed as means and standard deviations.

	Light	Moderate	Vigorous	MVPA^a^
*Reaction Flow*				
ActiGraph	3.57 (1.32)	2.73 (1.59)	0.73 (0.88)	3.46 (2.25)
Polar	2.39 (2.11)	2.20 (2.59)	0.62 (1.42)	2.81 (3.45)
*Whack A Mole*				
ActiGraph	3.76^b^ (1.40)	2.21 (1.64)	0.55 (0.87)	2.77 (2.31)
Polar	1.81^b^ (1.76)	3.66 (3.49)	0.48 (1.18)	4.14 (3.96)
*Super Hits (Catch*)				
ActiGraph	3.61 (1.68)	1.42 (1.57)	0.12 (0.17)	1.53 (1.71)
Polar	2.37 (2.00)	1.70 (2.29)	0.73 (1.83)	2.43 (3.41)
*Super Hits (Slash*)				
ActiGraph	4.03 (1.53)	1.15 (1.57)	0.08 (0.11)	1.23^c^ (1.65)
Polar	3.08 (1.84)	2.34 (2.33)	0.75 (1.97)	3.09^c^ (3.28)
Overall average				
ActiGraph	3.74^d^ (0.21)	1.88 (0.73)	0.37 (0.32)	2.25 (1.05)
Polar	2.41^d^ (0.52)	2.47 (0.84)	0.65 (0.12)	3.12 (0.73)

a MVPA: moderate-to-vigorous physical activity

b *P*=.005

c *P*=.02

d *P*=.003

Notably, Polar recorded higher MVPA times for most games compared to ActiGraph, except for *Reaction Flow*. ActiGraph consistently recorded higher light PA levels, particularly for *Whack A Mole* (Light_ActiGraph_=3.76 [SD 1.40] versus Light_Polar_=1.81 [SD 1.76]; *P*=.005) and for the overall average (Light_ActiGraph_= 3.74 [SD 0.21] versus Light_Polar_=2.41 [SD 0.52]; *P*=.003). *Whack A Mole* had the highest MVPA per Polar, 4.14 (SD 3.96) minutes, while *Reaction Flow* had the highest MVPA per ActiGraph, 3.46 (SD 2.25) minutes. The difference between the two PA measurement devices was statistically significant only for *Super Hits* (*Slash*) (MVPA_ActiGraph_=1.23 [SD 1.65] versus MVPA_Polar_=3.09 [SD 3.28]; *P*=.02).

Table 3 presents the average time participants spent in different zones within the 8×8 ft space. The majority of gameplay time occurred in Zone 1 (4×4 ft), where participants spent an average of 30.01 (SD 2.14) minutes, accounting for 75% of total gameplay. Zone 2 (6×6 ft) was used moderately, with participants spending 9.15 (SD 1.80) minutes (23% of total gameplay), while Zone 3 (8×8 ft) was rarely used, with only 0.93 (SD 0.35) minutes (2%). This distribution suggests a strong tendency for participants to remain within the smallest zone, highlighting the feasibility of playing these AAR games in constrained spaces, making them particularly suitable for home environments. Additionally, the limited movement across zones suggests that balance, rather than extensive mobility, can be more critical to gameplay, thereby lowering spatial barriers to engaging in PA.

**Table 3.** Duration spent in different zones in minutes, expressed as means and standard deviations.

	Reaction Flow	Whack a Mole	Super Hits (Catch)	Super Hits (Slash)	Total duration	% of time spent
Zone 1: 4×4 ft	4.30 (4.14)	8.68 (2.73)	8.39 (3.33)	8.63 (3.15)	30.00 (2.14)	75
Zone 2: 6×6 ft	4.97 (3.66)	1.16 (2.18)	1.64 (3.34)	1.39 (3.15)	9.15 (1.80)	23
Zone 3: 8×8 ft	0.75 (1.63)	0.18 (0.67)	0.00 (0.00)	0.00 (0.00)	0.93 (0.35)	2
Total	10.02 (2.27)	10.02 (4.65)	10.03 (4.45)	10.02 (4.64)	40.00 (0.10)	100

### Heart Rate and Rate of Perceived Exertion

All HR values collected during the protocol (in both BPM and %HRmax) reflect averages calculated from second-by-second readings. Before cognitive testing, participants’ HR averaged 75.75 (SD 12.85) BPM, with minimal exertion (RPE=0.12, SD 0.5). After the initial test, HR decreased to 72.25 (SD 12.32) BPM when RPE rose modestly to 0.43 (SD 0.72). Prior to gameplay, HR remained stable at 72.31 (SD 12.89) BPM, with a slight RPE increase to 0.62 (SD 0.71).

During gameplay, HR rose significantly to an average of 100 (SD 14.11) BPM, with post-gameplay RPE immediately increasing to 3.25 (SD 1.73), indicating moderate-to-high physical exertion. This converts to an average of 64.1% HR_Max_ during the gameplay, indicating that the gameplay on average elicited moderate PA [67]. The gameplay phase lasted approximately 40 minutes. In addition, participants rested for an average of 8.73 minutes (SD 3.70, range 2‐25) between each game to allow for HR recovery.

Before the post-cognitive test, HR remained somewhat elevated at 80.31 (SD 14.75) BPM with an RPE of 1.62 (SD 1.74), indicating lingering physical exertion effects. The RAs ensured that participants’ HR dropped within 10 BPM of resting HR before initiating the cognitive test. After the test, HR returned closer to baseline at 76 (SD 13.24) BPM and RPE decreased to 1.42 (SD 1.96). See Table 4 for details.

**Table 4.** Heart rate and rate of perceived exertion, expressed as means and standard deviations.

Sequence and variables	Value
Pre-cog tests	
Age-predicted maximum HR^a^ (HR_Max)_	156.01 (2.63)
Before pre-cog HR (BPM^b^)	75.75 (12.85)
Before pre-cog %HR_Max_	48.55
Before pre-cog RPE^c^ (scale)	0.12 (0.5)
After pre-cog HR (BPM)	72.25 (12.32)
After pre-cog %HR_Max_	46.31
After pre-cog RPE (scale)	0.43 (0.72)
Before gameplay	
HR (BPM)	72.31 (12.89)
Before gameplay %HR_Max_	46.34
RPE (scale)	0.62 (0.71)
During gameplay	
Duration (mins)	40.00 (0.10)
HR during Game 1 (BPM)	100.00 (14.76)
%HR_Max_ during Game 1 (%)	64.10
HR during Game 2 (BPM)	100.01 (14.96)
%HR_Max_ during Game 2 (%)	64.10
HR during Game 3 (BPM)	100.25 (14.35)
%HR_Max_ during Game 3 (%)	64.26
HR during Game 4 (BPM)	98.69 (14.71)
%HR_Max_ during Game 4 (%)	63.26
HR per game (BPM)	100.00 (14.11)
%HR_Max_ during all gameplay (%)	64.10
Resting time for HR recovery (min)	8.73 (3.70)
After gameplay	
HR (BPM)	98.87 (16.82)
After gameplay %HR_Max_	63.37
RPE (scale)	3.25 (1.73)
Post-cog tests	
Resting time for HR recovery (min)	7.26 (3.77)
Before post-cog HR (BPM)	80.31 (14.75)
Before post-cog %HR_Max_	51.48
Before post-cog RPE (scale)	1.62 (1.74)
After post-cog HR (BPM)	76 (13.24)
After post-cog %HR_Max_	48.71
After post-cog RPE (scale)	1.42 (1.96)

a HR: heart rate

b BPM: beats per minute.

c RPE: rate of perceived exertion.

Notably, the beta-blocker group consistently exhibited lower HR throughout the study—approximately 8 BPM less on average—compared to the non-taker group. Given that continuous RPE reporting during gameplay would have been disruptive, RPE was measured immediately after each game, following prior literature [80-82]. Additionally, they reported higher post-gameplay RPE than nontakers, though neither the HR nor RPE differences reached statistical significance due to the small sample sizes (7 takers vs 9 nontakers). Nevertheless, the mean values of average %HR_Max_ suggest that those not on beta-blockers generally met the moderate-intensity threshold during games, whereas those on beta-blockers tended to fall slightly below the moderate intensity threshold. As such, the data were presented in aggregate below (see Multimedia Appendix 3 for a breakdown between the two groups).

### Cognitive Tests

 The cognitive assessment, including the Flanker and visual reasoning tests, was conducted pre- and post-AAR gameplay to evaluate potential cognitive benefits. Visual reasoning scores improved significantly post-play (*P*=.04) in change-sensitive score (CSS), an item response theory–based score metric designed for longitudinal change monitoring. Starting from NIH Toolbox V3, age- and education-adjusted *T* scores are also provided [83]. The effect remained significant when adjusted by age alone and age and education combined (*P*’s = .03). See Table 5 for details. These results may suggest that visual reasoning abilities benefited from brief single-bout AAR gameplay, possibly due to the interactive, visually stimulating nature of the activities. However, an alternative explanation is that the improvement may be attributed to a practice effect on the task. To distinguish between these two possibilities, future studies should incorporate a control group to isolate the specific cognitive effects of AAR interventions.

**Table 5.** Cognitive tests (Flanker and visual reasoning) before and after AAR^a^ Play, expressed as means and standard deviations.

Cognitive test	Before	After	*T*	*P*
Flanker CSS^b^	503.81 (19.17)	501.81 (15.39)	0.76	.23
Flanker age adjusted standard score	105.69 (21.98)	103.44 (17.60)	0.73	.24
Flanker age edu adjusted standard score	53.50 (14.89)	51.94 (11.81)	0.73	.24
Visual reasoning CSS^b^	499.5 (8.52)	502.69 (10.66)	1.94^c^	.04^c^
Visual reasoning age adjusted standard score	102.88 (17.17)	109.31 (21.89)	1.98^c^	.03^c^
Visual reasoning age edu adjusted standard score	50.44 (10.01)	54.25 (12.08)	1.99^c^	.03^c^

a AAR: active augmented reality

b CSS: change-sensitive score

c *P*<.05

### Questionnaire Results

Table 6 summarizes participants’ responses across scales measuring AAR/PA motivation, intention, and game engagement. The AAR/PA intention score was 4.15 (SD 0.59) on a 5-point scale, reflecting a strong intention to continue engaging in AAR activities. Similarly, the AAR/PA motivation scale had a high mean score of 5.67 (SD 1.24) on a 7-point scale, suggesting participants were motivated to continue playing AAR games. In the GEQ subscales (5-point scale), all subscales except flow had an acceptable Cronbach α (≥0.6) and were averaged. Participants reported high positive affect, 4.35 (SD 0.80), and minimal negative affect, 1.30 (SD 0.46), with low tension, 1.25 (SD 0.67), suggesting that gameplay was generally enjoyable and nonstressful. They also reported relatively high competence 3.68 (SD 0.92) and sensory and imaginative immersion 3.64 (SD 0.88), showing that they felt capable and engaged during gameplay. The challenge level was moderate at 2.49 (SD 0.76), reflecting a manageable level of difficulty.

**Table 6.** Survey results for the AAR^a^/PA^b^ intention, motivation, and Game Experience Questionnaire (GEQ) measures, expressed as means and standard deviations^c^.

Survey result	Mean (SD)	Cronbach α
AAR /PA intention	4.15 (0.59)	.86
AAR/PA motivation	5.67 (1.24)	.96
GEQ
Competence	3.68 (0.92)	.88
Sensory and imaginative immersion	3.64 (0.88)	.81
Flow	—	.43
Tension	1.25 (0.67)	.86
Challenge	2.49 (0.76)	.67
Negative affect	1.30 (0.46)	.83
Positive affect	4.35 (0.80)	.82

a AAR: active augmented reality

b PA: physical activity

c All scales are 5-point scales except for AAR/PA motivation, which is a 7-point scale.

### Interview Analysis

 We used a thematic analysis approach to examine the qualitative interview data. Two team members independently reviewed the transcripts and conducted open coding to identify initial patterns. Codes were refined through discussion, and themes were inductively developed to reflect key participant perceptions of AAR gameplay, such as enjoyment, accessibility, physical challenge, and suggestions for improvement.

The thematic analysis revealed that older adult participants generally responded positively to the AR games, viewing them as both enjoyable and beneficial for combining physical exercise with mental engagement. Many appreciated the opportunity to play at home, describing it as an accessible and refreshing alternative to traditional exercise settings. While some initially had reservations about the novelty of AR or the perceived cost of setup, these concerns diminished with familiarity. Additionally, participants expressed a desire for more physically exerting games than those provided. Among the games played, *Whack a Mole* emerged as a favorite, with participants appreciating its simplicity and engaging nature. The interactive experience, particularly the combination of music and movement, was highlighted as a factor that fostered focus and enjoyment.

Looking ahead to future game design, participants expressed interest in customizable games that start at an easier level and progressively increase in difficulty, accommodating a range of physical abilities. Many suggested incorporating full-body movements and immersive environments, such as outdoor simulations, to further enhance engagement. Music integration, particularly with familiar tunes, was another highly requested feature. Beyond individual play, participants saw great potential for these games in supporting physical and mental health, particularly for older adults or individuals with limited mobility, ensuring a user-friendly interface for those less familiar with technology and incorporating community or group-based settings to foster social engagement and support.

## Discussion

### Principal Findings

This feasibility study highlights the potential of AAR games to increase PA and enhance cognitive function among older adults in a user-friendly and safe environment. Participants engaged in MVPA during gameplay, as measured by accelerometers and HR monitors, aligning with the recommended PA levels for older adults. Cognitive assessments revealed significant improvements in visual reasoning, indicating potential cognitive benefits from a single bout of AAR gameplay. Surveys indicated high levels of motivation, enjoyment, and positive emotions associated with AAR gameplay, with participants appreciating its accessibility and suitability for free-living environments. Interview feedback emphasized the need for adaptable game designs to cater to diverse physical abilities and individual preferences. Importantly, despite their various health conditions, all participants successfully completed the study protocol, underscoring the feasibility and acceptability of AAR for the older adult population.

### Comparison to Prior Research

Our findings revealed differences in PA measurement between devices, with the HR monitor, Polar, recording slightly higher MVPA levels compared to the accelerometer, ActiGraph. Since HR-based measurements offer a more individualized measurement of PA intensity, MVPA estimates from Polar may provide a more appropriate reflection of exertion levels, whereas ActiGraph measures may be more appropriate on a group but not individual level [84,85]. However, given that 7 out of 16 participants were taking beta-blockers, their actual MVPA level may have been higher than what the Polar has recorded, as beta-blockers can suppress HR responses to physical exertion [79]. These variations in PA measurement emphasize the need for careful selection of appropriate tracking methods in future AAR studies among older adults, particularly when accounting for medication effects on HR-based PA assessments.

Beyond the benefits afforded by the PA engagement, AAR gameplay also improved cognitive function, with significant gains in visual reasoning. This suggests that the AAR games may enhance cognitive processes related to problem-solving and cognitive flexibility. These results align with prior research linking PA to enhanced executive function, indicating that AAR games could stimulate brain regions associated with higher-order cognition and promote neural engagement [59,86,87]. While these effects may be attributed to AAR gameplay, the possibility of a practice effect on cognitive testing cannot be ruled out. Future studies should incorporate control groups to better isolate the specific cognitive effects of AAR interventions.

One of the most notable advantages of AAR gameplay is its accessibility and practicality for home-based exercise. Participants remained engaged and motivated, as reflected by their high AAR/PA intention and motivation scores, along with strong positive affect and immersion ratings. With initial training and a play environment designed to simulate the home environment, participants were able to engage in PA with minimal equipment and space requirements. Notably, the majority of the gameplay occurred within a 4×4 area (Zone 1), highlighting AAR’s feasibility in confined spaces and its potential to reduce barriers to PA among those with limited access to outdoor exercise spaces. Additionally, since AAR gameplay relies more on balance than extensive mobility, it offers a viable exercise option for individuals with physical limitations [28].

The qualitative findings further support these conclusions, with participants expressing enthusiasm for AAR games as a viable alternative to traditional exercise. Many appreciated the interactive nature of the games, particularly the integration of music and movement, and welcomed the flexibility of playing in a home-like environment. While some initially faced challenges with gameplay mechanics, their confidence improved over time, and they expressed interest in continued gameplay. Participants also suggested various customization options, such as adjustable difficulty levels, which could enhance long-term engagement and accommodate physical abilities.

### Limitations

 This study has several limitations. First, the small sample size (n=16), along with the relatively short duration, reduces statistical power and limits the generalizability of the findings, warranting larger-scale studies conducted over a longer period. Future research should aim to recruit a more representative sample. Second, 7 out of 16 participants were taking beta blockers, which may have influenced HR-based PA assessments and potentially led to underestimation of MVPA levels, as reflected in the lower %HR_max_ observed among takers compared to nontakers. Future research should explore alternative PA measurement methods or incorporate personalized adjustments for HR thresholds in individuals taking beta blockers. Although alternative PA measurement strategies or individualized adjustments to HR thresholds should be explored, to date, no widely accepted method exists for adjusting HR-based PA estimates among beta-blocker users. A larger sample and auxiliary measures such as RPE with a larger sample in both groups might help shed light on the PA intensity levels. Third, the lack of a control group prevents us from drawing causal conclusions regarding cognitive improvements from AAR gameplay. Future studies should include appropriate comparison conditions, such as passive and active control groups, to better isolate the cognitive effects of AAR interventions and strengthen causal inferences.

### Future Directions

Given the feasibility-focused nature of the study, we intentionally selected games with more moderate than vigorous PA intensities to prioritize safety. Despite this, several participants expressed a desire for more intense PA options, indicating that older adults may benefit from progressively vigorous AAR gameplay as they grow more comfortable with technology and gradually increase their PA levels. Although appropriate for a feasibility trial, this constrained intensity range may have limited the extent of cognitive changes observed, particularly if higher-intensity PA is a necessary condition for eliciting measurable cognitive benefit, though careful precautions are needed to ensure that high-intensity exercise is not potentially dangerous for older adults who are not regular exercisers. Future research should explore adaptive difficulty settings to allow for gradual progression in PA intensity, accommodating users at different fitness levels. In addition, future studies should examine a wider variety of AAR game types, longer intervention periods, and group-based gameplay to explore potential social and motivational benefits.

### Conclusions

Using a mixed-methods one-group pretest-posttest experimental design, the study demonstrated that older adults could engage in MVPA during AAR gameplay and showed significant improvements in visual reasoning. Participants reported high levels of motivation, enjoyment, and positive affect, and qualitative feedback further supported the acceptability and potential benefits of AAR interventions. These findings underscore the need for further exploration of long-term AAR interventions with larger, controlled trials to evaluate the sustained effects and optimize design features. With continued development and refinement, AAR games may serve as a viable and acceptable intervention tool for promoting PA and cognitive engagement among older adults.

## Supplementary material

10.2196/73221 Multimedia Appendix 1 Online questionnaire.

10.2196/73221 Multimedia Appendix 2 Screenshots of the visual reasoning (Up) and the Flanker inhibitory control and attention tests (Down).

10.2196/73221 Multimedia Appendix 3 Heart rate and rate of perceived exertion between beta-blocker nonusers and users, expressed as means and standard deviations.

## Abbreviations

AAR: active augmented reality
AR: augmented reality
AVG: active video game
BPM: beats per minute
GEQ: Gaming Experience Questionnaire
HR: heart rate
HRR: heart rate reserve
IGNITE: Investigating Gains in Neurocognition in an Intervention Trial of Exercise
MVPA: moderate-to-vigorous physical activity
PA: physical activity
RA: research assistants
RPE: rate of perceived exertion
RQ: research question
VM: vector magnitude
VR: virtual reality

## References

[1] Vincent GK, Velkoff VA (2010). The next four decades: the older population in the united states: 2010 to 2050. https://www.census.gov/content/dam/Census/library/publications/2010/demo/p25-1138.pdf.

[2] Rowe JW, Kahn RL (2015). Successful aging 2.0: conceptual expansions for the 21st century. J Gerontol B Psychol Sci Soc Sci.

[3] Milanović Z, Pantelić S, Trajković N, Sporiš G, Kostić R, James N (2013). Age-related decrease in physical activity and functional fitness among elderly men and women. Clin Interv Aging.

[4] Suryadinata RV, Wirjatmadi B, Adriani M, Lorensia A (2020). Effect of age and weight on physical activity. J Public Health Res.

[5] Becofsky K, Baruth M, Wilcox S (2016). Physical activity mediates the relationship between program participation and improved mental health in older adults. Public Health (Fairfax).

[6] Musich S, Wang SS, Hawkins K, Greame C (2017). The frequency and health benefits of physical activity for older adults. Popul Health Manag.

[7] Soares-Miranda L, Siscovick DS, Psaty BM, Longstreth WT, Mozaffarian D (2016). Physical activity and risk of coronary heart disease and stroke in older adults: the cardiovascular health study. Circulation.

[8] Logsdon RG, Gibbons LE, McCurry SM, Teri L (2002). Assessing quality of life in older adults with cognitive impairment. Psychosom Med.

[9] Mosti CB, Rog LA, Fink JW, Ravdin LD, Katzen HL (2019). Handbook on the Neuropsychology of Aging and Dementia.

[10] Murman DL (2015). The Impact of Age on Cognition. Semin Hear.

[11] Hertzog C, Kramer AF, Wilson RS, Lindenberger U (2008). Enrichment effects on adult cognitive development: can the functional capacity of older adults be preserved and enhanced?. Psychol Sci Public Interest.

[12] Pedone C, Ercolani S, Catani M (2005). Elderly patients with cognitive impairment have a high risk for functional decline during hospitalization: the GIFA study. J Gerontol A Biol Sci Med Sci.

[13] Diehl M, Willis SL, Schaie KW (1995). Everyday problem solving in older adults: observational assessment and cognitive correlates. Psychol Aging.

[14] (2015). Transforming our world: the 2030 agenda for sustainable development. United Nations Department of Economic and Social Affairs.

[15] Blumenthal JA, Smith PJ, Mabe S (2019). Lifestyle and neurocognition in older adults with cognitive impairments. Neurology (ECronicon).

[16] Brasure M, Desai P, Davila H (2018). Physical activity interventions in preventing cognitive decline and Alzheimer-type dementia: a systematic review. Ann Intern Med.

[17] Downey A, Stroud C, Landis S, Leshner AI, National Academies of Sciences, Engineering, and Medicine (2017). Preventing Cognitive Decline and Dementia: A Way Forward.

[18] Petersen RC (2016). Mild cognitive impairment. Continuum (Minneap Minn).

[19] Halloway S, Arfanakis K, Wilbur J, Schoeny ME, Pressler SJ (2019). Accelerometer physical activity is associated with greater gray matter volumes in older adults without dementia or mild cognitive impairment. J Gerontol B Psychol Sci Soc Sci.

[20] Stubbs B, Eggermont L, Soundy A, Probst M, Vandenbulcke M, Vancampfort D (2014). What are the factors associated with physical activity (PA) participation in community dwelling adults with dementia? A systematic review of PA correlates. Arch Gerontol Geriatr.

[21] Buchner DM, Beresford SA, Larson EB, LaCroix AZ, Wagner EH (1992). Effects of physical activity on health status in older adults. II. Intervention studies. Annu Rev Public Health.

[22] Pandey A, Garg S, Khunger M (2015). Dose-response relationship between physical activity and risk of heart failure: a meta-analysis. Circulation.

[23] Ribas TM, Teodori RM, Mescolotto FF, Montebelo M, Baruki SBS, Pazzianotto-Forti EM (2021). Impact of physical activity levels on musculoskeletal symptoms and absenteeism of workers of a metallurgical company. Rev Bras Med Trab.

[24] Schuch FB, Vancampfort D, Rosenbaum S (2016). Exercise for depression in older adults: a meta-analysis of randomized controlled trials adjusting for publication bias. Braz J Psychiatry.

[25] (2022). Physical activity. World Health Organization.

[26] Suzuki Y, Maeda N, Hirado D, Shirakawa T, Urabe Y (2020). Physical activity changes and its risk factors among community-dwelling Japanese older adults during the COVID-19 epidemic: associations with subjective well-being and health-related quality of life. Int J Environ Res Public Health.

[27] König M, Gollasch M, Komleva Y (2023). Frailty after covid‐19: the wave after?. Aging Med (Milton).

[28] Rúa-Alonso M, Bovolini A, Costa-Brito AR (2023). Exploring perceived barriers to physical activity among older adults living in low-population density regions: gender differences and associations with activity dimensions. Healthcare (Basel).

[29] Newsome AM, Batrakoulis A, Camhi SM (2024). 2025 ACSM worldwide fitness trends: future directions of the health and fitness industry. ACSM’s Health Fitness J.

[30] Margrett JA, Ouverson KM, Gilbert SB, Phillips LA, Charness N (2022). Older adults’ use of extended reality: a systematic review. Front Virtual Real.

[31] Zhao MY, Ong SK, Nee AYC (2016). An augmented reality-assisted therapeutic healthcare exercise system based on bare-hand interaction. Int J Human Comput Interact.

[32] (2022). Top augmented reality statistics in 2022. Art labs.

[33] Augumented reality market by device type, offering, application, technology, and geography. Marketsandmarkets.com.

[34] (2022). America’s interest in virtual & augmented reality. ARRIS Composites.

[35] (2023). 95% of americans eager to embrace AR/VR technology, but point to price and health concerns as top barriers. AddictiveTips Staff.

[36] Malik SA, Abdullah LM, Mahmud M, Azuddin M Presented at: 2013 international conference on research and innovation in information systems (ICRIIS); Nov 27-28, 2013; Kuala Lumpur, Malaysia. 2013.[doi: 10.1109/ICRIIS.2013.6716739].

[37] Guerrero Huerta AG, Hernández Rubio E (2017). Interaction Modalities for Augmented Reality in Tablets for Older Adults.

[38] Derby JL, Chaparro BS (2020). Human Aspects of IT for the Aged Population Technologies, Design and User Experience.

[39] Anderson M, Perrin A (2017). Tech Adoption Climbs Among Older Adults.

[40] Ku J, Kim YJ, Cho S, Lim T, Lee HS, Kang YJ (2019). Three-dimensional augmented reality system for balance and mobility rehabilitation in the elderly: a randomized controlled trial. Cyberpsychol Behav Soc Netw.

[41] Jeon S, Kim J (2020). Effects of augmented-reality-based exercise on muscle parameters, physical performance, and exercise self-efficacy for older adults. Int J Environ Res Public Health.

[42] Rohrbach N, Gulde P, Armstrong AR (2019). An augmented reality approach for ADL support in Alzheimer’s disease: a crossover trial. J Neuroeng Rehabil.

[43] Espay AJ, Baram Y, Dwivedi AK (2010). At-home training with closed-loop augmented-reality cueing device for improving gait in patients with Parkinson disease. J Rehabil Res Dev.

[44] Goumopoulos C, Drakakis E, Gklavakis D (2023). Feasibility and acceptance of augmented and virtual reality exergames to train motor and cognitive skills of elderly. Computers.

[45] Lee LN, Kim MJ, Hwang WJ (2019). Potential of augmented reality and virtual reality technologies to promote wellbeing in older adults. Appl Sci (Basel).

[46] Chen M, Tang Q, Xu S, Leng P, Pan Z (2020). Design and evaluation of an augmented reality-based Exergame system to reduce fall risk in the elderly. IJERPH.

[47] Philippe AG, Goncalves A, Korchi K, Deshayes M (2024). Exergaming in augmented reality is tailor-made for aerobic training and enjoyment among healthy young adults. Front Public Health.

[48] Erickson KI, Grove GA, Burns JM (2019). Investigating gains in neurocognition in an intervention trial of exercise (IGNITE): protocol. Contemp Clin Trials.

[49] Oberlin LE, Wan L, Kang C (2025). Cardiorespiratory fitness is associated with cognitive function in late adulthood: baseline findings from the IGNITE study. Br J Sports Med.

[50] Warburton DE, Jamnik VK, Bredin SS, Gledhill N (2011). The physical activity readiness questionnaire for everyone (PAR-Q+) and electronic physical activity readiness medical examination (ePARmed-X+). Health Fitness J Canada.

[51] Haley SM, Jette AM, Coster WJ (2002). Late life function and disability instrument: II. Development and evaluation of the function component. J Gerontol A Biol Sci Med Sci.

[52] (2021). Active arcade ios. https://apps.apple.com/us/app/active-arcade/id1553158383.

[53] (2021). Active Arcade Android.

[54] (2022). Apple announces winners of the 2022 apple design awards. Apple Inc.

[55] (2022). Active arcade. Nex.

[56] Morishita S, Tsubaki A, Nakamura M, Nashimoto S, Fu JB, Onishi H (2019). Rating of perceived exertion on resistance training in elderly subjects. Expert Rev Cardiovasc Ther.

[57] Weintraub S, Dikmen SS, Heaton RK (2013). Cognition assessment using the NIH Toolbox. Neurology (ECronicon).

[58] Colcombe S, Kramer AF (2003). Fitness effects on the cognitive function of older adults: a meta-analytic study. Psychol Sci.

[59] Kramer AF, Hahn S, Cohen NJ (1999). Ageing, fitness and neurocognitive function. Nature New Biol.

[60] Hwang J, Lu AS (2018). Narrative and active video game in separate and additive effects of physical activity and cognitive function among young adults. Sci Rep.

[61] McDonough DJ, Pope ZC, Zeng N, Lee JE, Gao Z (2018). Comparison of college students’ energy expenditure, physical activity, and enjoyment during exergaming and traditional exercise. J Clin Med.

[62] Strath SJ, Swartz AM, Bassett DR, O’Brien WL, King GA, Ainsworth BE (2000). Evaluation of heart rate as a method for assessing moderate intensity physical activity. Med Sci Sports Exerc.

[63] Hwang J, Fernandez AM, Lu AS (2018). Application and validation of activity monitors’ epoch lengths and placement sites for physical activity assessment in exergaming. J Clin Med.

[64] Aguilar-Farias N, Peeters G, Brychta RJ, Chen KY, Brown WJ (2019). Comparing ActiGraph equations for estimating energy expenditure in older adults. J Sports Sci.

[65] Sasaki JE, John D, Freedson PS (2011). Validation and comparison of ActiGraph activity monitors. J Sci Med Sport.

[66] Tanaka H, Monahan KD, Seals DR (2001). Age-predicted maximal heart rate revisited. J Am Coll Cardiol.

[67] American College of Sports Medicine (2021). ACSM’s Guidelines for Exercise Testing and Prescription.

[68] Miller NE, Strath SJ, Swartz AM, Cashin SE (2010). Estimating absolute and relative physical activity intensity across age via accelerometry in adults. J Aging Phys Act.

[69] Troiano RP, Berrigan D, Dodd KW, Mâsse LC, Tilert T, Mcdowell M (2008). Physical activity in the united states measured by accelerometer. Med Sci Sports Exe.

[70] Puterman E, Lin J, Blackburn E, O’Donovan A, Adler N, Epel E (2010). The power of exercise: buffering the effect of chronic stress on telomere length. PLoS ONE.

[71] Chudasama YV, Khunti KK, Zaccardi F (2019). Physical activity, multimorbidity, and life expectancy: a UK Biobank longitudinal study. BMC Med.

[72] (2023). Cognition measures. NIH Toolbox.

[73] Rhodes RE, Macdonald HM, McKay HA (2006). Predicting physical activity intention and behaviour among children in a longitudinal sample. Soc Sci Med.

[74] Kendzierski D, DeCarlo KJ (1991). Physical activity enjoyment scale: two validation studies. J Sport Exerc Psychol.

[75] IJsselsteijn WA, Kort YA, Poels K The game experience questionnaire. https://pure.tue.nl/ws/files/21666907/Game_Experience_Questionnaire_English.pdf.

[76] Lu AS, Buday R, Thompson D, Baranowski T, Tettegah S, Huang WD (2016). Emotions, Technology, and Digital Games.

[77] Lu AS, Green MC, Thompson D (2019). Using narrative game design to increase children’s physical activity: exploratory thematic analysis. JMIR Serious Games.

[78] George D, Mallery P (2019). IBM SPSS Statistics 26 Step by Step: A Simple Guide and Reference: Routledge.

[79] Schrack JA, Leroux A, Fleg JL (2018). Using heart rate and accelerometry to define quantity and intensity of physical activity in older adults. J Gerontol A Biol Sci Med Sci.

[80] Christen J, Foster C, Porcari JP, Mikat RP (2016). Temporal robustness of the session rating of perceived exertion. Int J Sports Physiol Perform.

[81] Ferreira SS, Krinski K, Alves RC (2014). The use of session RPE to monitor the intensity of weight training in older women: acute responses to eccentric, concentric, and dynamic exercises. J Aging Res.

[82] Foster C, Florhaug JA, Franklin J (2001). A new approach to monitoring exercise training. J Strength Cond Res.

[83] Where are the fully-corrected scores in NIH toolbox V3. NIH Toolbox cognition measures.

[84] Evenson KR, Buchner DM, Morland KB (2012). Objective measurement of physical activity and sedentary behavior among US adults aged 60 years or older. Prev Chronic Dis.

[85] Matthew CE (2005). Calibration of accelerometer output for adults. Med Sci Sports Exerc.

[86] Choi E, Shin SH, Ryu JK (2021). Association of extensive video gaming and cognitive function changes in brain-imaging studies of pro gamers and individuals with gaming disorder: systematic literature review. JMIR Serious Games.

[87] Hötting K, Röder B (2013). Beneficial effects of physical exercise on neuroplasticity and cognition. Neurosci Biobehav Rev.

