# Supplementary material for: The Impact of Active Augmented Reality Games on Physical Activity and Cognition Among Older Adults: Feasibility Study
Source: JMIR Serious Games. 2025 Oct 3;13:e73221. doi: 10.2196/73221 (PMC12494185; doi:10.2196/73221)
Supplement: Multimedia Appendix 1 [file games-v13-e73221-s001.docx]

Appendix 1: Online Questionnaire

1. AAR / PA Intention (Disagree = 1; Neutral = 3; Agree = 5)

1. Being physically active every day for the next 3 months would be fun.
2. Being physically active every day for the next 3 months would be enjoyable.
3. Being physically active every day for the next 3 months would be good for me.
4. Being physically active every day for the next 3 months would be important for me.
5. People close to me want me to be physically active every day over the next 3 months.
6. People who are important to me want me to be physically active every day over the next 3 months.
7. My doctors want me to be physically active every day over the next 3 months.
8. People close to me will be physically active every day over the next 3 months.
9. People who are important to me will be physically active every day over the next 3 months.
10. My doctors will be physically active every day over the next 3 months.
11. I could be physically active every day over the next 3 months if I really wanted to.
12. I have the time to be physically active every day over the next 3 months if I wanted to.
13. I have a place to be physically active every day over the next 3 months if I wanted to.
14. I plan to be physically active every day over the next 3 months.
15. I intend to be physically active every day over the next 3 months.

2. AAR / PA Motivation: Please choose **ONE** number that is closer to the statement.

| 1. I enjoy it. | 1 2 3 4 5 6 7 | I hate it. |
| --- | --- | --- |
| 1. I feel bored. | 1 2 3 4 5 6 7 | I feel interested. |
| 1. I dislike it. | 1 2 3 4 5 6 7 | I like it. |
| 1. I find it pleasurable. | 1 2 3 4 5 6 7 | I find it unpleasurable. |
| 1. I am very absorbed in this activity. | 1 2 3 4 5 6 7 | I am not at all absorbed in this activity. |
| 1. It is no fun at all. | 1 2 3 4 5 6 7 | It is a lot of fun. |
| 1. I find it energizing. | 1 2 3 4 5 6 7 | I find it tiring. |
| 1. It makes me depressed. | 1 2 3 4 5 6 7 | It makes me happy. |
| 1. It is very pleasant. | 1 2 3 4 5 6 7 | It is very unpleasant. |
| 1. I feel good physically while doing it. | 1 2 3 4 5 6 7 | I feel bad physically while doing it. |
| 1. It’s very invigorating. | 1 2 3 4 5 6 7 | It’s not at all invigorating. |
| 1. I am very frustrated by it. | 1 2 3 4 5 6 7 | I am not at all frustrated by it. |
| 1. It’s very gratifying. | 1 2 3 4 5 6 7 | It’s not at all gratifying. |
| 1. It’s very exhilarating. | 1 2 3 4 5 6 7 | It’s not at all exhilarating. |
| 1. It’s not at all stimulating. | 1 2 3 4 5 6 7 | It’s very stimulating. |
| 1. It gives me a strong sense of accomplishment. | 1 2 3 4 5 6 7 | It does not give me any sense of accomplishment. |
| 1. It’s very refreshing | 1 2 3 4 5 6 7 | It’s not at all refreshing |
| 1. I felt as though I would rather be doing something else | 1 2 3 4 5 6 7 | I felt as though there was nothing else I would rather be doing |

3. AAR Gaming Experience Questionnaire (GEQ) (Not at all = 1; Slightly =2; Moderately = 3; Fairly = 4; Extremely =5)

1. I felt content.
2. I felt skillful.
3. I was interested in the game's story.
4. I thought it was fun.
5. I was fully occupied with the game.
6. I felt happy.
7. It gave me a bad mood.
8. I thought about other things.
9. I found it tiresome.
10. I felt competent.
11. I thought it was hard.
12. It was aesthetically pleasing.
13. I forgot everything around me.
14. I felt good.
15. I was good at it.
16. I felt bored.
17. I felt successful.
18. I felt imaginative.
19. I felt that I could explore things.
20. I enjoyed it.
21. I was fast at reaching the game's targets.
22. I felt annoyed.
23. I felt pressured.
24. I felt irritable.
25. I lost track of time.
26. I felt challenged.
27. I found it impressive.
28. I was deeply concentrated in the game.
29. I felt frustrated.
30. It felt like a rich experience.
31. I lost connection with the outside world.
32. I felt time pressure.
33. I had to put a lot of effort into it.

4. Social Desirability (Disagree = 1; Neutral = 3; Agree = 5)

1. I like everyone I know.
2. I am always kind.
3. I always have good manners.
4. I am always good.
5. I am always nice to everyone.
6. I tell the truth every single time.
7. I never get angry.
8. I never say things I shouldn’t.
9. I never lie.
