# Supplementary material for: The Impact of Active Augmented Reality Games on Physical Activity and Cognition Among Older Adults: Feasibility Study
Source: JMIR Serious Games. 2025 Oct 3;13:e73221. doi: 10.2196/73221 (PMC12494185; doi:10.2196/73221)

Appendix 2: Screenshots of the Visual Reasoning (Up) and the Flanker Inhibitory Control and Attention Tests (Down)


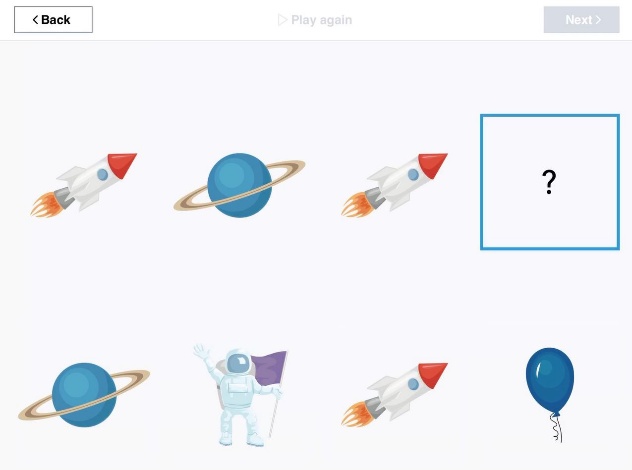


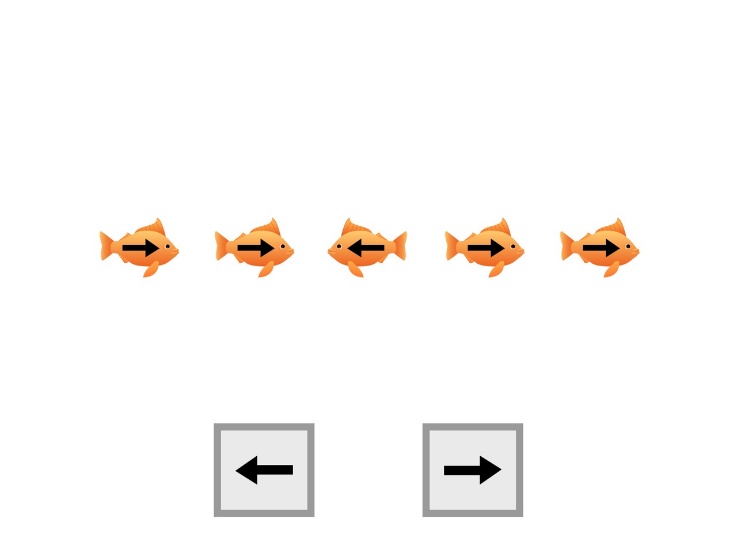

Supplement: Multimedia Appendix 2 [file games-v13-e73221-s002.docx]
