# Supplementary material for: The Impact of Active Augmented Reality Games on Physical Activity and Cognition Among Older Adults: Feasibility Study
Source: JMIR Serious Games. 2025 Oct 3;13:e73221. doi: 10.2196/73221 (PMC12494185; doi:10.2196/73221)
Supplement: Multimedia Appendix 3 [file games-v13-e73221-s003.docx]

Appendix 3. Heart Rate and Rate of Perceived Exertion between Beta-blocker non-users and users, expressed as means and standard deviations

| Sequence | Variable | Non-Users (n=9) | Users (n=7) |
| --- | --- | --- | --- |
| Pre-Cog tests | Age-predicted maximum HR | 157.01 (2.92) | 154.8 (1.62) |
|  | Before pre-cog HR (BPM) | 80.67 (10.77) | 69.42 (13.24) |
|  | Before pre-cog %HR_Max_ | 51.38 | 44.84 |
|  | Before pre-cog RPE (scale) | 0.22 (0.67) | 0 (0) |
|  | After pre-cog HR (BPM) | 75.22 (13.53) | 68.43 (10.26) |
|  | After pre-cog %HR_Max_ | 47.91 | 44.21 |
|  | After pre-cog RPE (scale) | 0.56 (0.88) | 0.29 (0.49) |
| Before Gameplay | HR (beats/ min) | 75.78 (14.22) | 67.86 (10.25) |
|  | Before gameplay %HR_Max_ | 48.26 | 43.84 |
|  | RPE (scale) | 0.78 (0.83) | 0.43 (0.53) |
| During Gameplay | Duration (mins) | 39.99 (0.03) | 40.00 (0.15) |
|  | HR during Game 1 (BPM) | 103.22 (13.20) | 95.71 (16.60) |
|  | %HR_Max_ during Game 1 (%) | 65.72 | 61.83 |
|  | HR during Game 2 (BPM) | 103 (13.80) | 96.29 (16.61) |
|  | %HR_Max_ during Game 2 (%) | 65.58 | 62.20 |
|  | HR during Game 3 (BPM) | 103.89 (13.62) | 95.57 (14.90) |
|  | %HR_Max_ during Game 3 (%) | 66.15 | 61.74 |
|  | HR during Game 4 (BPM) | 101.33 (15.95) | 95.29 (13.34) |
|  | %HR_Max_ during Game 4 (%) | 64.52 | 61.55 |
|  | HR per game (BPM) | 102.86 (13.48) | 95.71 (14.89) |
|  | %HRMax during all gameplay (%) | 65.49 | 61.83 |
|  | Resting time for HR recovery (mins) | 8.63 (4.67) | 8.87 (2.56) |
| After Gameplay | HR (BPM) | 98.11 (18.71) | 99.86 (15.44) |
|  | After gameplay %HR_Max_ | 62.49 | 64.51 |
|  | RPE (scale) | 2.89 (1.62) | 3.71 (1.89) |
| Post-Cog tests | Resting time for HR recovery (mins) | 7.07 (3.34) | 7.50 (4.53) |
|  | Before post-cog HR (BPM) | 84.67 (14.56) | 74.71 (14.03) |
|  | Before post-cog %HR_Max_ | 53.92 | 48.26 |
|  | Before post-cog RPE (scale) | 1.44 (1.42) | 1.86 (2.19) |
|  | After post-cog HR (BPM) | 79.67 (13.62) | 76 (12.05) |
|  | After post-cog %HR_Max_ | 50.74 | 49.10 |
|  | After post-cog RPE (scale) | 0.89 (1.36) | 2.14 (2.48) |
